# Supplementary material for: Causal integration of multi‐omics data with prior knowledge to generate mechanistic hypotheses
Source: Mol Syst Biol. 2021 Jan 27;17(1):e9730. doi: 10.15252/msb.20209730 (PMC7838823; doi:10.15252/msb.20209730)
Supplement: Supplementary file 2 — Appendix [file MSB-17-e9730-s001.pdf]

Appendix figures for:

Causal integration of multi-omics data with prior knowledge to generate mechanistic hypotheses

Aurelien Dugourd[1,2,3,4†], Christoph Kuppe[3,4,†], Marco Sciacovelli[5†], Enio Gjerga[1,2], Attila Gabor[1], Kristina B. Emdal[6], Vitor Vieira[13], Dorte B. Bekker-Jensen[6], Jennifer Kranz[7,8], Eric. M. J. Bindels[9], Ana S. H. Costa[5,10], Abel Sousa[14,15], Pedro Beltrao[15], Miguel Rocha[13], Jesper V. Olsen[6], Christian Frezza[5,*], Rafael Kramann[3,411*], Julio Saez-Rodriguez[1,2,12,*]

[1] Heidelberg University, Faculty of Medicine, and Heidelberg University Hospital, Institute for Computational Biomedicine, Bioquant, Heidelberg, Germany
[2] RWTH Aachen University, Faculty of Medicine, Joint Research Centre for Computational Biomedicine (JRC-COMBINE), Aachen, Germany
[3] Institute of Experimental Medicine and Systems Biology, Faculty of Medicine, RWTH Aachen University, Aachen, Germany
[4] Division of Nephrology and Clinical Immunology, Faculty of Medicine, RWTH Aachen University, Aachen, Germany
[5] MRC Cancer Unit, University of Cambridge, Hutchison/MRC Research Centre, Cambridge, UK
[6] Proteomics Program, Novo Nordisk Foundation Center for Protein Research, Faculty of Health and Medical Sciences, University of Copenhagen, Copenhagen, Denmark
[7] Department of Urology and Pediatric Urology, St. Antonius Hospital Eschweiler, Academic Teaching Hospital of RWTH Aachen, Eschweiler, Germany
[8] Department of Urology and Kidney Transplantation, Martin Luther University, Halle (Saale), Germany
[9] Department of Hematology, Erasmus MC, Rotterdam, The Netherlands
[10] Current address : Cold Spring Harbor Laboratory, One Bungtown Road, Cold Spring Harbor, NY, USA
[11] Department of Internal Medicine, Nephrology and Transplantation, Erasmus Medical Center, Rotterdam, The Netherlands
[12] Molecular Medicine Partnership Unit, European Molecular Biology Laboratory and Heidelberg University, Heidelberg, Germany
[13] Centre of Biological Engineering, University of Minho - Campus de Gualtar, Braga, Portugal
[14] Institute for Research and Innovation in Health (i3s), Porto, Portugal
[15] European Molecular Biology Laboratory, European Bioinformatics Institute (EMBL-EBI), Wellcome Genome Campus, Hinxton, UK

Content:

Appendix note 1
Threshold choice rational
Appendix Figure S1
Coherence assessment between CARNIVAL hypotheses and underlying data.
Appendix Figure S2
COSMOS solution network from our patient samples.
Appendix Figure S3
Comparison of COSMOS network co-regulation predictions with data-driven co-regulations between kinases phosphatases and TFs.
Appendix Figure S4
Distribution of edge weight differences for network shuffling.
Appendix Figure S5
COSMOS solution network from breast cancer cell line data.

Appendix note 1

The COSMOS network solution aims at connecting a downstream layer (the measurements) with upstream regulators (the inputs). The choice of the cutoff will determine which inputs and measurements will be connected together by COSMOS. Thus, changing the cutoff has two main consequences with respect to the resulting COSMOS network.

First, if more (or less) inputs and measurements are provided, the network solution will contain additional (or fewer) edges to connect them.

Second, giving more (or less) inputs and measurements to COSMOS means giving more (or less) information to COSMOS to build a coherent network to connect them, making the problem easier (or harder) to solve.

Knowing that, the choice of the threshold has to be decided with respect to (i) which are the TFs, kinases, phosphatase and metabolites that a user wishes to potentially connect together (ii) how confident the user is that the TFs, kinases, phosphatase and metabolites are actually deregulated and (iii) how much information should be provided to COSMOS to find a coherent network connecting TFs, kinases, phosphatase and metabolites.

To illustrate this and show how the COSMOS network may change with respect to cutoff changes, we made three additional runs of COSMOS (connecting downstream metabolites with upstream TFs and kinases, that is the "forward" run). We chose (1) a very loose cutoff (p-value < 0.5 and absolute activity score > 0.6 sd, essentially including everything), (2) a cutoff reducing the number of upstream TFs and kinases used as upstream input while keeping the same measurements as the original COSMOS run (p-value < 0.05 and absolute activity score > 2.4 sd) and (3) a very stringent cutoff (p-value < 0.001 and absolute activity score > 5.2 sd).

As expected, the loose threshold yielded the largest network while the most stringent one yielded the smallest (200 edges cutoff (1), 108 edges for cutoff (2) and 50 edges for cutoff (3), compared the 162 edges of the original network). COSMOS also had more difficulty solving the problem when less TFs and kinases were given as upstream inputs (optimality gap = 8.17% for cutoff (2) compared to the original optimality gap = 2.36%). We compared the network of cutoff (2) with the original network (in the same manner as for the network shuffling analysis, see Material and Methods, Meta PKN contextualisation for explanation on the edge weight) and found that the solutions were relatively similar, with a median absolute weight difference of 25%.

To conclude, the cutoff choice depends on the situation. For example, we may be specifically interested in some TFs or metabolites, or we may want to give more importance to finding a large network connecting as many TFs, kinase and metabolites together (while being less confident regarding their actual deregulation).

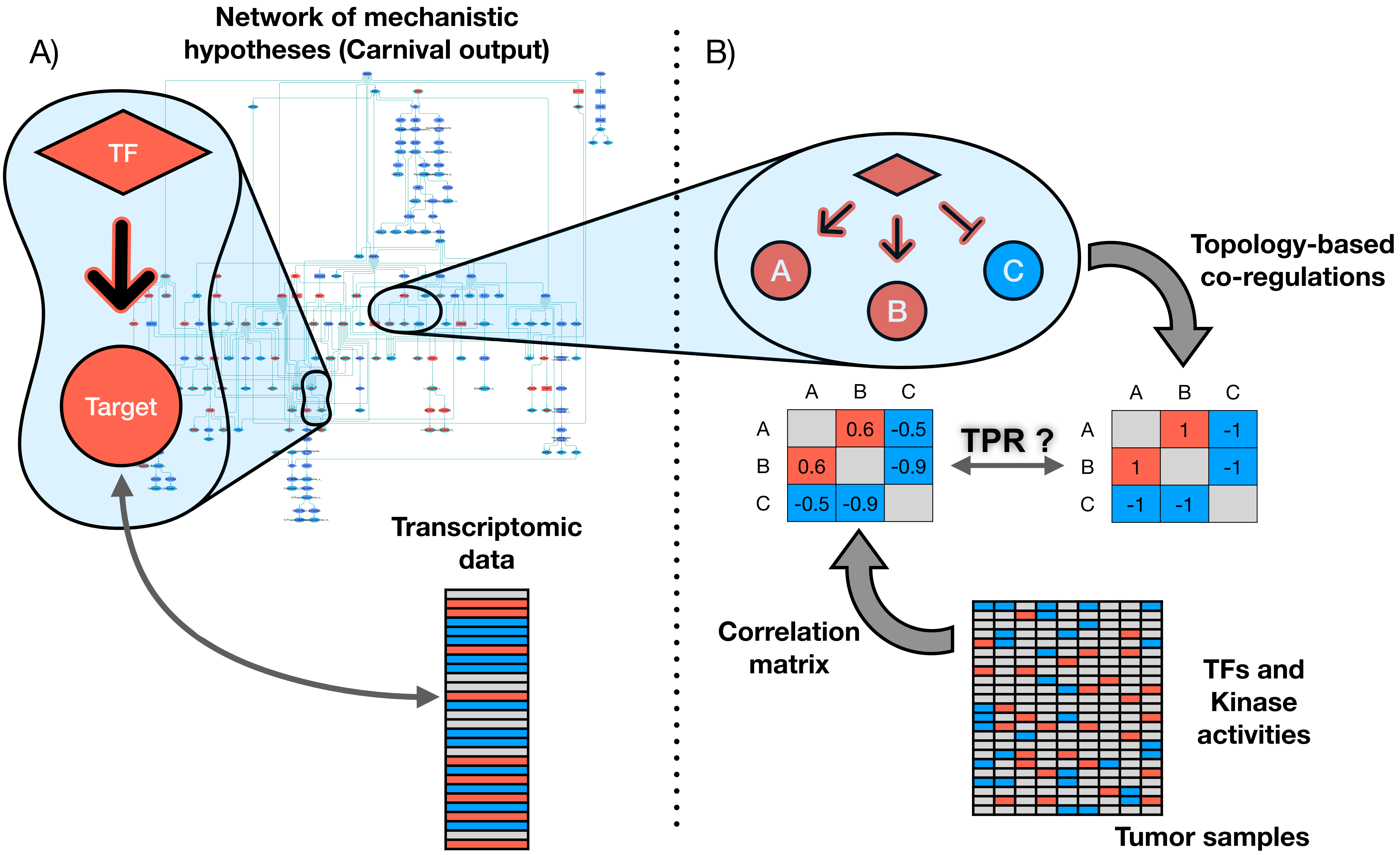

Appendix Figure S1
Coherence assessment between CARNIVAL hypotheses and underlying data. On the left, the predicted activity TF targets of the COSMOS network are compared to the actual t-value (tumor - healthy) of their corresponding transcript to filter incoherent interactions (correction step of the network pre-processing, see Meta PKN contextualisation). On the right, coregulations predicted by COSMOS are compared against a correlation network of kinase/ TF activities to determine TPR.

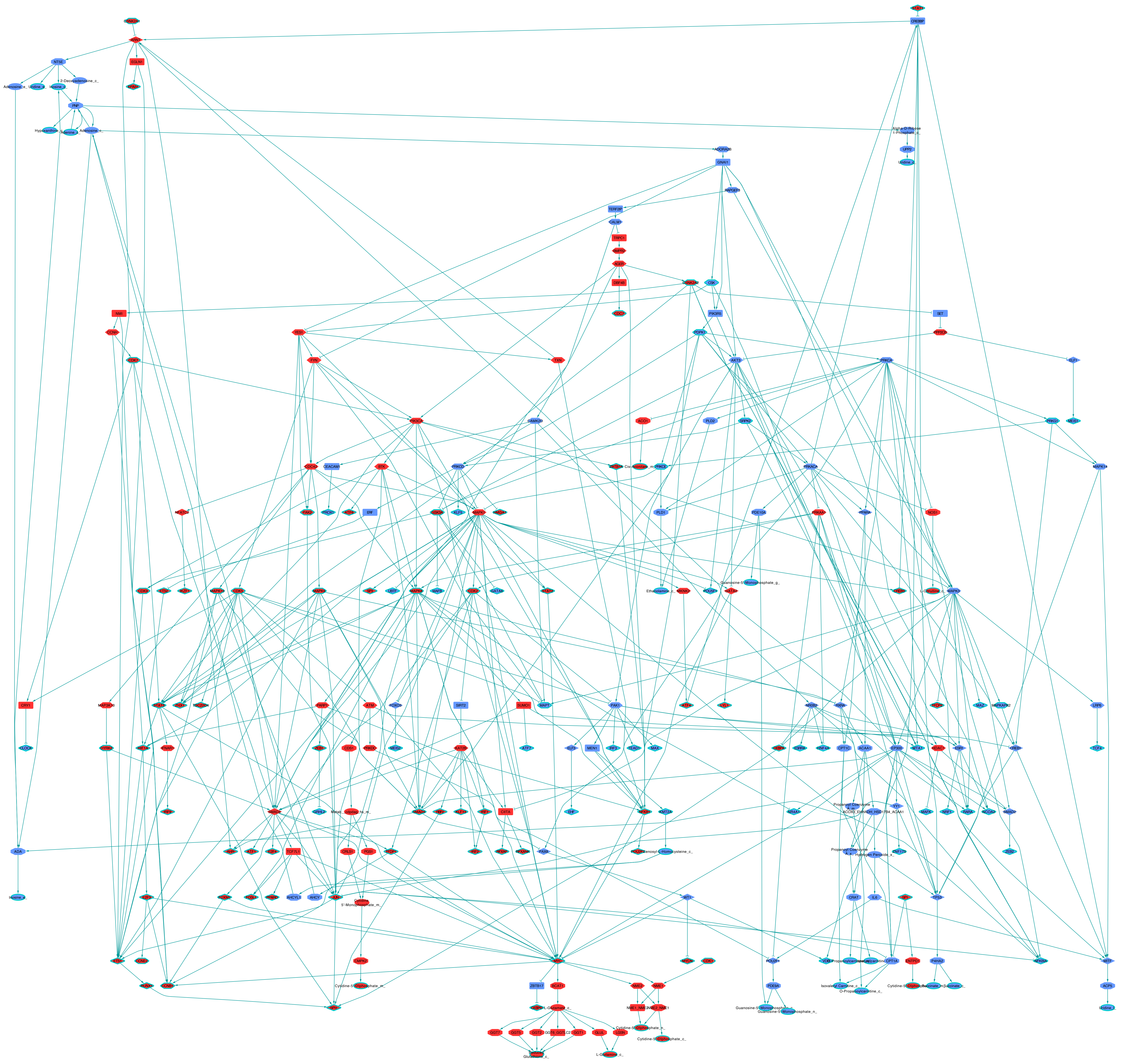

Appendix Figure S2

Causal network summarising the mechanistic hypotheses systematically generated by CARNIVAL. The network comprises 449 edges. It represents the propagation of signals connecting the deregulated kinases, phosphatases, TFs and metabolites in kidney cancer.

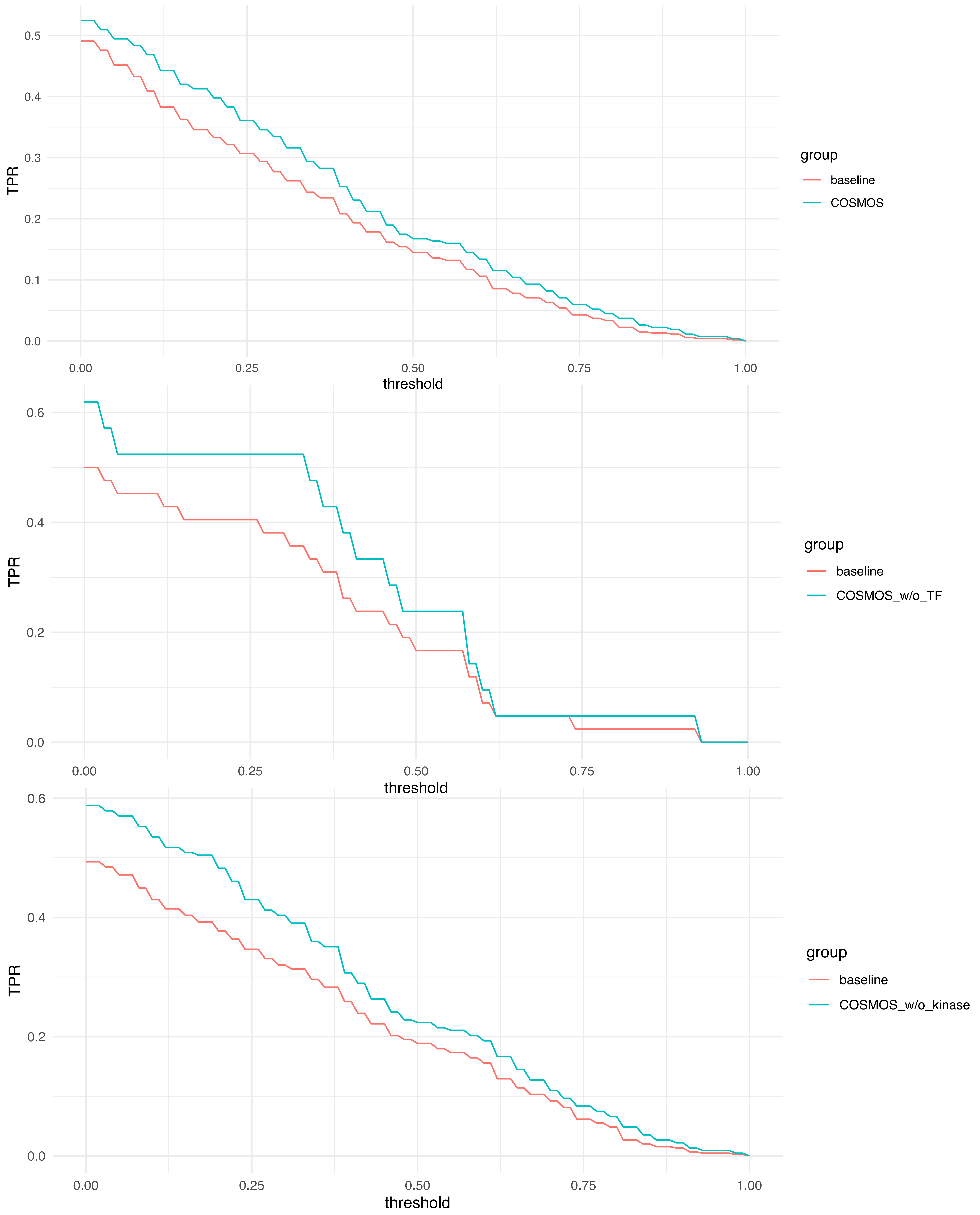

Appendix Figure S3

Comparison of COSMOS network co-regulation predictions with data-driven co-regulations between kinases phosphatases and TFs. Top panel shows the performance of COSMOS with all three omics layers. Middle panel shows performance when TFs are hidden. Bottom panel shows performance when kinases are hidden. Each panel compares the ability of COSMOS to capture co-regulation events between kinases/phosphatases and transcription factors that are consistent with observed correlations in the data itself.

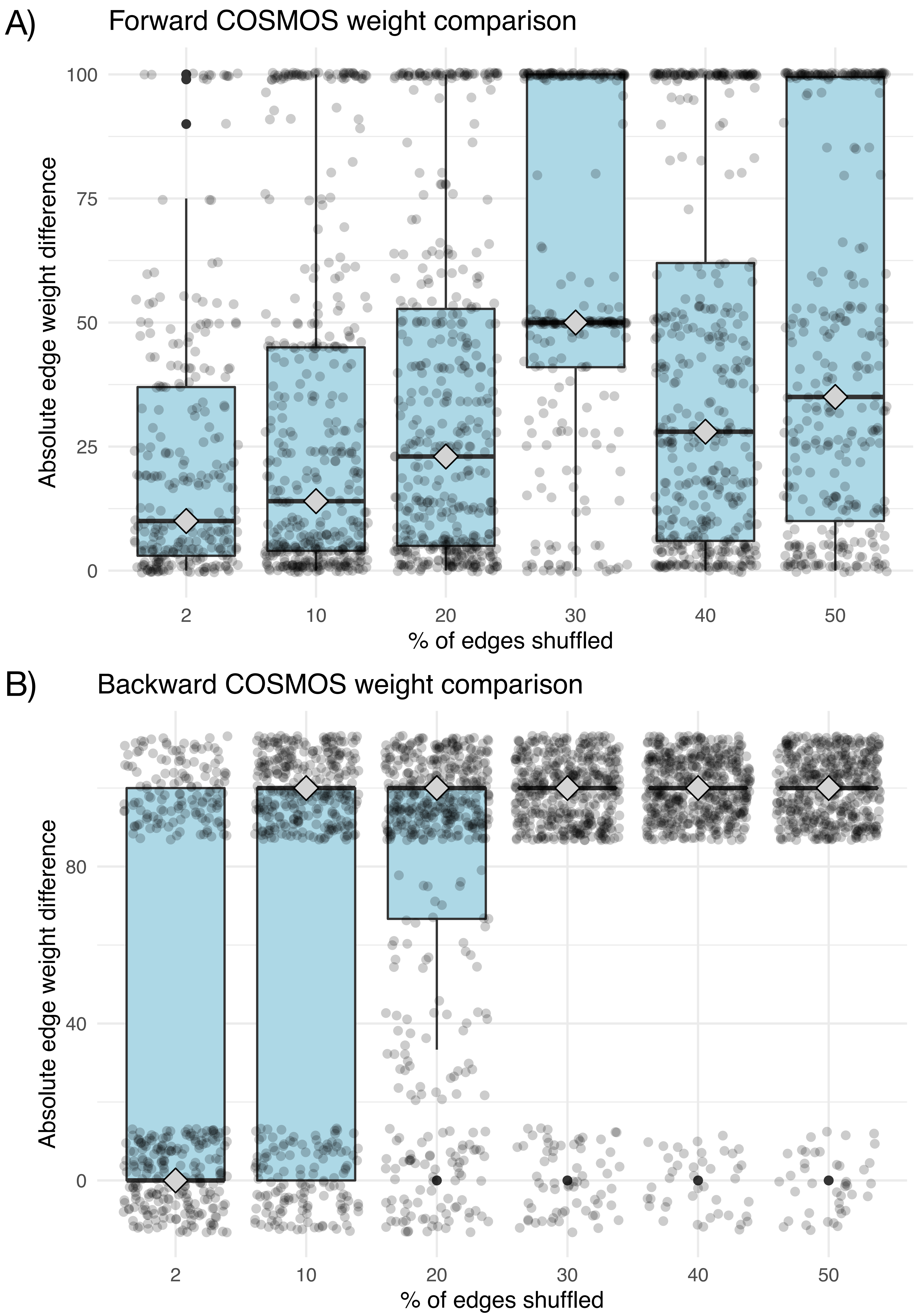

Appendix Figure S4
Distribution of edge weight differences between A) 'forward' and B) 'backward' results obtained from the original PKN and 2, 10, 20, 30, 40 and 50% shuffled PKNs. Each dot represents the absolute weight difference for a given edge. The diamonds represent the medians of the weight difference distributions. The boxes cover 25th to 75th percentiles of the distributions.

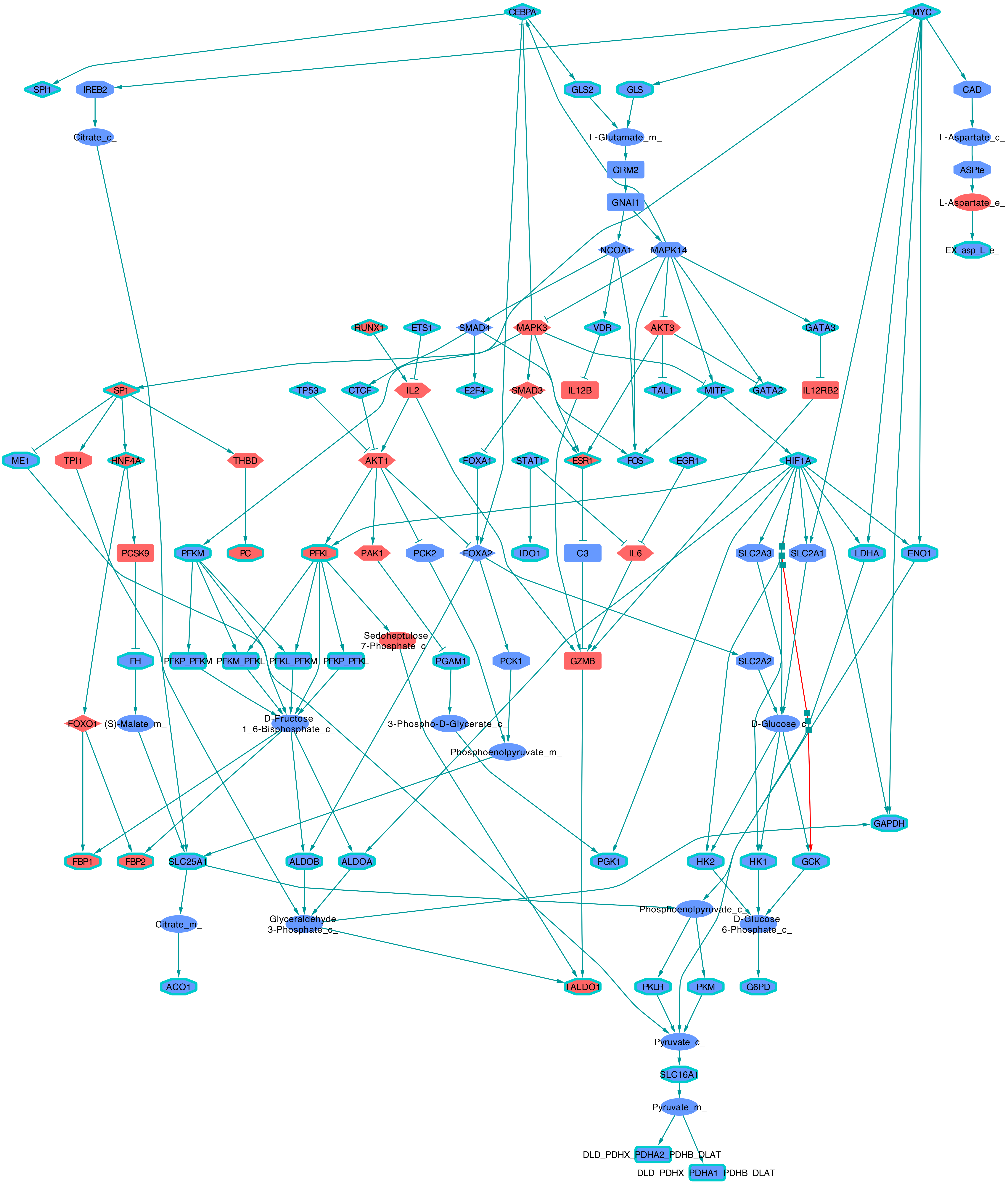

Appendix Figure S5

COSMOS solution network connecting metabolic fluxes and TF activity deregulations observed in a breast cancer cell line cultured with and without glutamine.
