# Supplementary material for: Causal integration of multi‐omics data with prior knowledge to generate mechanistic hypotheses
Source: Mol Syst Biol. 2021 Jan 27;17(1):e9730. doi: 10.15252/msb.20209730 (PMC7838823; doi:10.15252/msb.20209730)
Supplement: Supplementary file 3 — Expanded View Figures PDF [file MSB-17-e9730-s002.pdf]

Expanded View Figures

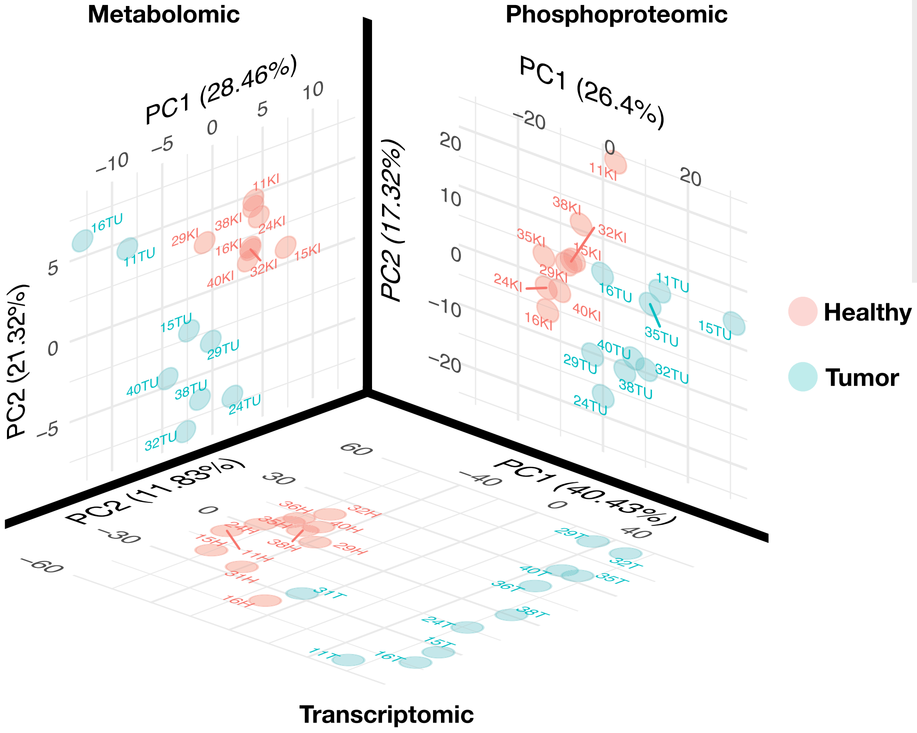

Figure EV1. First two components of PCA of omic datasets of kidney tumor and healthy samples.

PCA of metabolomics (107 metabolites), phosphoproteomics (14,243 phosphosites) and transcriptomics (15,919 transcripts) datasets for tumor and healthy tissues samples. For each omics dataset, PCA is run independently on normalised datasets and the first two components are plotted. Each omics shows a clear separation between tumor and healthy tissue.

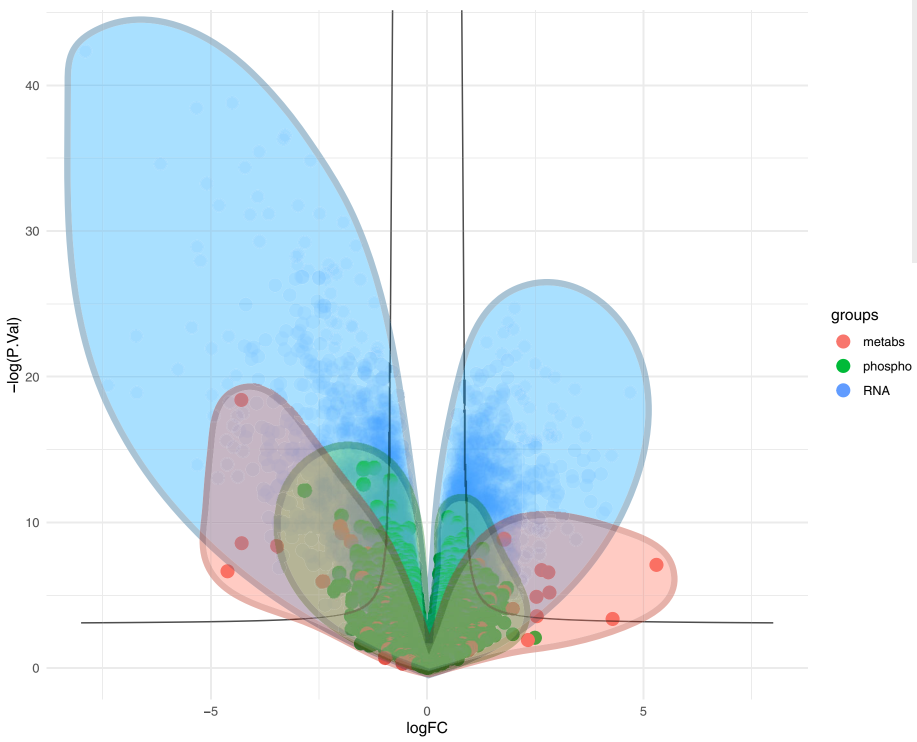

Figure EV2. Differential analysis of kidney tumor and healthy sample.

Combined volcano plot of metabolomics (107 metabolites), phosphoproteomics (14,243 phosphosites) and transcriptomics (15,919 transcripts), displaying the surface occupied by the points of each type of omic data. X axis represent the $\log_2$ fold change between healthy and tumour. *Y* axis represents the $-\log_{10}$ *P*-value of a unpaired moderated *t*-statistic obtained from using LIMMA.

© 2021 The Authors

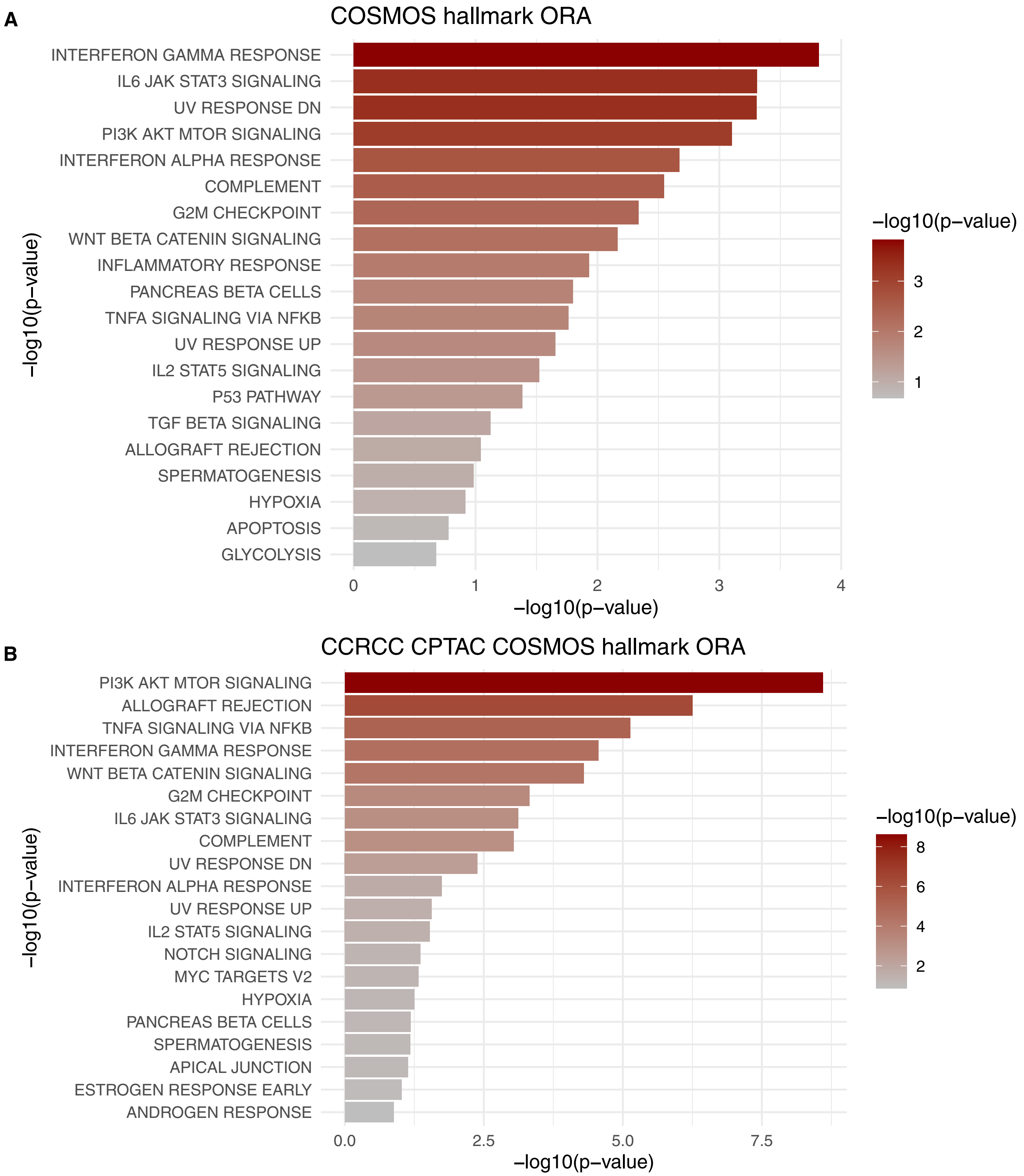

Figure EV3. Ranked over-represented pathways in COSMOS solution networks.

A, B Comparison of over-representation analysis performed with msigDB HALLMARK pathways between COSMOS networks generated from (A) our patient samples and (B) CPTAC patient cohort. Overall, a very similar set of pathways was significantly over-represented in both cases, notably PI3K-AKT-MTOR signaling, TNFA signaling via NFKB, interferon gamma response, WNT beta catenin signaling, G2M checkpoint and IL6 JAK STAT3 signaling.

© 2021 The Authors

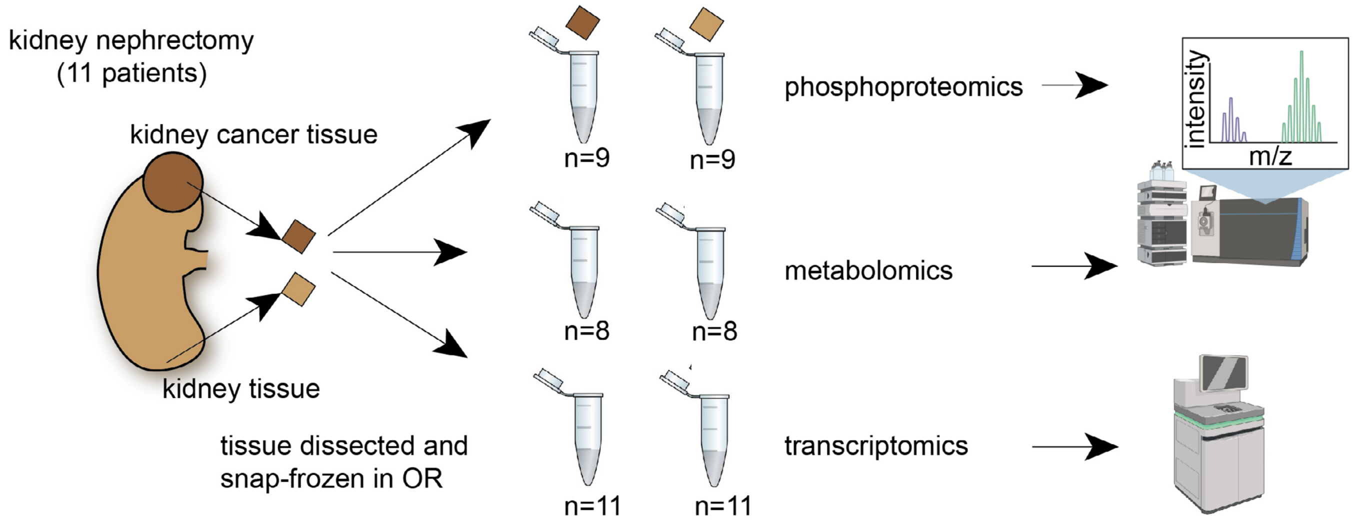

Figure EV4. Overview of sample collection and study design.

Schematic of the process sample collection and multi-omics data generation. We included a total of 22 samples from 11 renal cancer patients (6 men, age 65.0 ± 14.31, 5 women, age 65.2 ± 9.257(mean ± SD)) for transcriptomics. Phosphoproteomics was also measured in a subset of 18 samples from 9 of these patients (6 men, age 65 ± 14.31; 3 women, age 63.33 ± 11.06(mean ± SD)), and metabolomics was also measured in 16 samples from 8 out of these 9 patient (5 men, age 62 ± 13.23; 3 women, age 63.33 ± 9.89(mean ± SD), Dataset EV1. Patients underwent nephrectomy due to renal cancer. We processed tissue from within the cancer and a distant unaffected area of the same kidney. The tissue was snap-frozen immediately after nephrectomy within the operation room. Figure parts created with BioRender.com.

 © 2021 The Authors
